# Antibacterial and antioxidant properties of crude extract, fractions and compounds from the stem bark of *Polyscias fulva* Hiern (Araliaceae)

**DOI:** 10.1186/s12906-017-1572-z

**Published:** 2017-02-07

**Authors:** Guy Sedar Singor Njateng, Zhizhi Du, Donatien Gatsing, Raymond Simplice Mouokeu, Yaping Liu, Hong-Xia Zang, Jianlong Gu, Xiaodong Luo, Jules-Roger Kuiate

**Affiliations:** 1 Laboratory of Microbiology and Antimicrobial Substances, Faculty of Science, University of Dschang, P.O. Box 67, Dschang, Cameroon; 2 Institute of Fisheries and Aquatic Sciences, University of Douala, P.O Box 7236, Douala, Cameroon; 3 State Key Laboratory of Phytochemistry and Plant Resources in West China, Kunming Institute of Botany, Chinese Academy of Sciences, Kunming, 650204 People’s Republic of China

**Keywords:** *Polyscias fulva*, Compounds, Bacteria, Reactive molecules

## Abstract

**Background:**

In our previous work, the dichloromethane-methanol (1:1 v/v) extract, fractions and isolated compounds from *Polyscias fulva* stem bark showed interesting antifungal activity. As a continuity of that work, this study aimed to bring out complementary informations about the antimicrobial properties of *P. fulva* stem bark that may be useful in the standardization of phytomedicine from this plant.

**Methods:**

The antibacterial activities of the crude extract, fractions (*n*-hexane, ethyl acetate, *n*-butanol and residual) and isolated compounds from *Polyscias fulva* stem bark were assayed by broth microdilution techniques. Their antioxidant activity were evaluated using 2,2-diphenyl-1-picrylhydrazyl (DPPH), pyrogallol (superoxide anion) and β-carotene - linoleic acid assays.

**Results:**

The crude extract presented antibacterial activities against *S. typhi* (ATCC 6539), *E. aerogenes* (ATCC 13045), *P. aeruginosa* (PA01) and *E. coli* (ATCC 10536) with MIC values of 2000 to 8000 μg/ml. The fractionation led the ethyle acetate and *n*-butanol fractions relatively more active (MIC = 500 to 1000 μg/ml) as compared to the crude extract. *β*-sitosterol and 3-*O*-*α*-L- arabinopyranosyl-hederagenin were the most active compounds on the tested bacteria with MIC values ranging from 6.25 to 100 μg/ml. The most sensitive was *P. aeruginosa* (PA01) on which all the tested compounds were active with MICs ranging from 6.25 to 400 μg/ml. Among all the tested substances, the crude extract (RSa50 = 84.86 μg/ml) and the methyl atrarate (RSa50 = 14.77 μg/ml), showed the highest scavenging activities against DPPH free radicals and those arising from the oxidation of the linoleic acid respectively.

**Conclusion:**

From this study, the results obtained reveal that the stem bark of *P. fulva* possesses antibacterial and antioxidant activities. It may then be useful in the development of an antimicrobial phytomedicine with a large spectrum of actvity endowed with antioxidant properties which can be standardised based on the isolated compounds.

## Background

The frequency of life-threatening diseases due to pathogenic microorganisms has risen world-wide and is becoming a major cause of morbidity and mortality in immuno compromised patients [1]. This is due to the fact that microorganisms like bacteria nowadays tend to become resistant to drugs, coupled to the importunate side effects of some antibiotics. Furthermore, many infections due to microorganisms lead to the production of strongly reactive molecules from the oxygen metabolism [2] that can give rise to a variety of pathological conditions among which atherosclerosis, cardiovascular dysfunction, inflammation, carcinogenesis, reperfusion injury, drug toxicity and neurodegenerative diseases [3]. There is an urgent need to control microbial infections using appropriate antimicrobials devoided of side effects [4]. The World Health Organization [5] estimates that, in developing countries, about three quarters of the population rely on plant based traditional medicinal system. Plants such as vegetables and medicinal herbs constitute an important pool of molecules such as nitrogen compounds, phenolic compounds, terpenoids, vitamins and some other endogenous metabolites with free radical scavenging activities or antioxidant properties [6]. Many works have been carried out in order to discover new antimicrobial and antioxidant compounds from different sources such as animals, microorganisms and plants. But much still has to be done since our botanical flora is made up of thousands of unexploited medicinal plants [7]. Among these medicinal plants is *Polyscias fulva*, used in Cameroon against venereal infections [7]. Some biological activities of *Polyscias fulva* have been reported. In our previous studies, the dichloromethane-methanol (1:1 v/v) crude extract showed interesting antidermatophytic activities both in vitro and in vivo [8]. A number of biologically active (against fungi) compounds have been isolated from *P. fulva* including two phenolics, one steroid, one triterpene and seven terpenoid saponins [9]. However, to the best of our knowledge, no information on its antibacterial and free radical scavenging properties is available. So, this work was designed to bring out complementary informations about the antimicrobial properties of *P. fulva* stem bark that may be useful in the standardization of a phytomedicine.

## Methods

### Microorganisms

The antimicrobial activities of the tested substances were carried out on fifteen bacteria made up of two strains of Gram positive bacteria: *Staphylococcus aureus* (ATCC 25922) and *Enterococcus faecalis* (ATCC 10541)*,* nine strains of Gram negative bacteria: *Pseudomonas aeruginosa* (PA01), *Pseudomonas aeruginosa* (ATCC 27853)*, Escherichia coli* (ATCC 8739)*, Escherichia coli* (ATCC 10536)*, Escherichia coli* (ATCC 11775), *Enterobacter aerogenes* (ATCC 13048), *Klepsiella pneumoniae* (ATCC13883), *Providencia stuartii* (ATCC 29916), *Salmonella typhi* (ATCC 6539) and four clinical isolates of gram negative bacteria: *Salmonella parathyphi* A*, Salmonella paratyphi* B*, Shigella flexneri* and *Proteus mirabilis.*

The references strains ATCC and the clinical isolates were obtained from the American Type Culture Collection (Rockville, MD, USA) and the Laboratory of Bacteriology and Mycology of the “Centre Pasteur” of Yaoundé-Cameroon respectively. These microorganisms were maintained on agar slant in refrigerator at 4 °C.

### Phytochemical materials

**Fig. 1:**
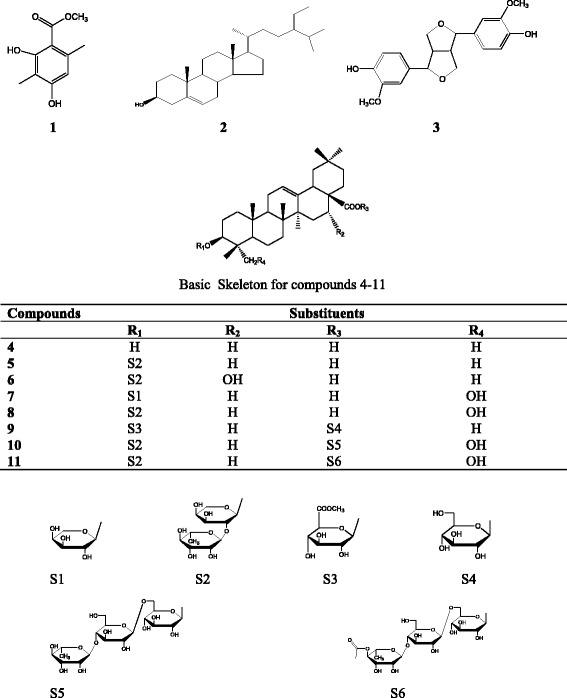
Chemical structures of compounds isolated from the stem barks of *P. fulva*

The tested materials were obtained during our previous work and included the dichloromethane-methanol (1:1 v/v) extract, the *n*-hexane, ethyl acetate, *n*-butanol and residual fractions, as well as compounds (Fig. 1): methyl 2,4-dihydroxy-3,6-dimethylbenzoate (Methyl atrarate) (**1**), *β*-sitosterol (**2**), pinoresinol (**3**), oleanolic acid (**4**), 3-*O*-[*α*-L-rhamnopyranosyl (1–2)-*α*-L-arabinopyranosyl]-oleanolic acid or *β*-hederagenin (**5**), 3-*O*-[*α*-L-rhamnopyranosyl (1–2)-*α*-L-arabinopyranosyl]-echinocystic acid (**6**), 3-*O-α*-L- arabinopyranosyl-hederagenin (**7**), 3-*O*-[*α*-L-rhamnopyranosyl (1–2)-*α*-L-arabinopyranosyl]-hederagenin (**8**), 3-*O*-[methyl-*β*-D-glucurono-pyranosiduronoate]-28-*O-β*-D-glucopyranosyl oleanolate (**9**), 3-*O*-[α-L-rhamnopyranosyl (1–2)-*α*-L-arabinopyranosyl]-28-*O*-[*O*-*α*-L-rhamnopyranosyl (1–4)-*O-β*-D-glucopyranosyl-(1–6)-*β*-D-glucopyranosyl]-hederagenin (**10**), 3-*O*-[*α*-L-rhamnopyranosyl (1–2)-*α*-L-arabinopyranosyl]-28-*O*-[*α*-L-4-*O*-acetyl-rhamnopyranosyl (1–4)-*β*-D-glucopyranosyl-(1–6)-*β*-D-glucopyranosyl]-hederagenin (**11**) [9].

### In vitro antibacterial assay

#### Preparation and standardisation of inocula

Inocula of bacteria were prepared separately from 24 h Muller Hinton agar cultures. From these cultures, colonies from each microorganism were diluted in 0.9% NaCl to obtain a turbidity matching the 0.5 of Mc Farland standard turbidity scale corresponding to about 1.5x10^8^ colony forming unit (CFU) per ml. The microbial suspensions were diluted to match the optical density of 0.1 at 600 nm (Jenway 6105UV/Vis spectrophotometer, 50Hz/60Hz) corresponding to about 10^6^ CFU/ml [10].

#### The broth microdilution test

The broth microdilution method [11] was used to determine the minimum inhibitory concentration (MIC) and minimum Bactericidal Concentration (MBC) of the tested substances using 96-well microplates (Nunclon, Roskilde, Danmark).

The 96-well plates were prepared by introducing into each well 100 μl of Mueller Hinton Broth (MHB). Into the first wells of the microplate was added 100 μl of each test substance followed by a serial two-fold dilutions of these test samples. A volume of 100 μl of the standardized inocula was then added into each well to match approximately 5 x 10^5^ CFU/ml in a total volume of 200 μl. This gave a final concentration range of 8 to 0.0625 mg/ml for extract, 1 to 0.0078 mg/ml for fractions and 0.1 to 0.00078 mg/ml for compounds as well as reference drug (Rifampicin). For every experiment, sterility control (broth and 5% v/v aqueous DMSO) and negative controls made up of broth plus inoculum on one hand and 5% v/v aqueous DMSO, broth plus inoculums on other hand were included. The content of each well was mixed thoroughly and the microplates were covered with the sterile lids and incubated at 37 °C for 24 h on a plate shaker (Flow Laboratory, Germany) at 300 rpm. After incubation, bacterial growth was monitored colorimetrically using iodonitrotetrazolium chloride (INT, Sigma-Aldrich, South Africa). MIC was the lowest concentration of the test substances that prevented visible growth of the microorganisms.

The minimum bactericidal concentration (MBC) values were determined by subculturing 50 μl aliquots of the preparations, which did not show any visible growth of the bacteria during MIC determinations, into 150 μl of test sample-free MHB. These preparations were further incubated as indicated above. Bacterial growth in each well was determined as mentioned above. MBC was the lowest concentration of the test samples that prevented visible growth of the bacteria in the sub-cultures. All the experiments were handled in triplicates.

### Antioxydant activity assay

Many types of reactive molecules can be produced in the organism, reason why three different methods were used for evaluating the scavenging activity.

#### DPPH assay

Radical scavenging activity of extract and compounds were determined using the stable free radical, 1,1-diphenyl-2-picrylhydrazyl (DPPH) by DPPH radical scavenging activity assay [12]. All the test substances and DPPH (20 mg/l) free radical were dissolved in methanol. The crude extract solution was prepared at 2 mg/ml, the isolated compounds at 1 mg/ml and L-ascorbic acid (reference drug) at 0.32 mg/ml. Two-fold serial dilutions were made to obtain the concentration ranging from 200 to 12.5 μg/ml, 100 to 6.25 μg/mland 32 to 2 μg/mlfor plant extract, compounds and the reference substance respectively. DPPH radical solution was prepared daily and the mixtures were made by adding 100 μl of test sample solution or reference substance to 900 μl of DPPH radical solution in a spectrophotometric vat. The content was mixed and the absorbance read immediately (t = 0 min) at 517 nm and then incubated at room temperature. After 30 min of incubation, the absorbance was recorded. All the tests were carried out in triplicate. The absorbance of the mixture of test solution/reference substance (100 μl) plus methanol (900 μl) were equally recorded. The percentages of DPPH radical reduction by test samples were calculated using the following formula:

$$ \mathrm{I}\%=\left[\left(\mathrm{Absorbance}\ \mathrm{of}\ \mathrm{DPPH}{\textstyle \hbox{-}}\mathrm{Absorbance}\ \mathrm{of}\ \mathrm{mixture}\right)/\mathrm{Absorbance}\ \mathrm{of}\ \mathrm{DPPH}\right]\ \mathrm{x}\ 100 $$

Where: Mixture = Extract, fraction or compound + methanolic solution of DPPH.

#### Superoxide anion scavenging activity

The method of Ekanayake et al. [13] was used in this test. The method is based on the inhibition of the auto-oxidation of pyrogallol by phenolic compounds. To the assay mixture made up of 2.6 ml of phosphate buffer solution (50 mM in water, pH 8.22 ± 0.03) and 0.3 ml of the analytical sample extract was added 0.1 ml of a freshly prepared solution of pyrogallol (3 mM in 0.01 M HCl). The auto-oxidation reaction rate of pyrogallol was determined at 325 nm by monitoring the absorbance for a total period of 10 min, corresponding to the end of the reaction. The scavenging activity of the superoxide anion (O^−2^) was calculated using the following formula:

$$ \mathrm{S}=\left[\left(\mathrm{K}0-\mathrm{K}1\right)/\mathrm{K}0\right]\mathrm{x}100 $$

Where K0 and K1 are the auto-oxidation rates of the pyrogallol without and with the extracts, or products, respectively.

#### β-Carotene/linoleic acid assay

In this assay, the antioxidant capacity of each test sample was determined by measuring the inhibition of the volatile organic compounds and the conjugated diene hydroperoxides arising from the linoleic acid oxidation. The method described by Tepe et al. [14] was used with a slight modification. In fact, a stock solution of *β*-Carotene and linoleic acid was prepared with 0.5 mg of *β*-carotene in 1 ml of chloroform, 25 μl of linoleic acid and 200 mg of Tween 40. The chloroform was evaporated under vacuum and 100 ml of aerated distilled water was then added to the residue. 300 μl of extract, fraction and compound was added to 2.5 ml of the previous mixture. The samples were dissolved in DMSO. The contents of testtubes were incubated in hot water (50 °C) for 2 h, together with two blanks, one containing the antioxidant ascorbic acid as a positive control and the other with the same volume of DMSO instead of the test sample. In the test tube with ascorbic acid, the yellow colour was maintained during the incubation period. The absorbance was measured at 470 nm. Antioxidant capacities (inhibition percentage, I %) of the tested samples were calculated using the following equation:

$$ \mathrm{I}\% = \left(\mathrm{Initial}\beta -\mathrm{Carotene}\ \mathrm{content}-\beta -\mathrm{Carotene}\ \mathrm{content}\ \mathrm{after}\ 2\ \mathrm{h}\ \mathrm{assay}/\mathrm{initial}\beta -\mathrm{Carotene}\ \mathrm{content}\right)\ \mathrm{x}\ 100 $$

Tests were carried out in triplicate. Percent inhibitions of the samples were compared with that of the positive standards.

### Statistical analysis

The probits of the radical scavenging percentages were plotted against the logarithmic values of concentration of test samples and a linear regression curve (Probit = f (log_10_ c)) was established in order to calculate the RSa50, which is the amount of sample necessary to decrease the free radicals by 50%. The obtained data were subjected to the one-way analysis of variance (ANOVA) and the results were expressed where appropriate as mean ± standard deviation. Differences between means of samples were compared using Duncan’s multiple range tests at *P* < 0.05.

## Results

### Antibacterial activities of the extract, fractions and isolated compounds

#### Antibacterial properties of the extract and fractions

Antibacterial properties were detected in crude extract and the fractions. These properties were generally very low and were observed on selected bacteria (Table 1). The fractionation led the ethyle acetate and *n*-butanol fractions relatively more active (MIC = 500 to 1000 μg/ml) as compared to the crude extract (MIC = 2000 to 8000 μg/ml) (Table 1).

**Table 1:** MICs and MBCs (μg/ml) of the crude dichloromethane-methanol (1:1 v/v) extract and fractions of *P. fulva* stem bark on tested bacterial strains

Bacteria	C E	n-hex F	EA F	n-but F	Res F	Rif
	MIC/MBC	MIC/MBC	MIC/MBC	MIC/MBC	MIC/MBC	MIC/MBC
Gram (-)						
*P. aeruginosa* (PA01)	8000/-	-/-	-/-	-/-	-/-	0.19/1.56
*P. aeruginosa* (ATCC 27853)	-/-	-/-	-/-	-/-	-/-	25/50
*E. coli* (ATCC 8739)	-	-/-	-/-	-/-	-/-	0.09/6.25
*E. coli* (ATCC 10536)	8000/-	-/-	-/-	-/-	-/-	0.19/0.78
*E. coli* (ATCC 11775)	-/-	8000/-	500/-	-/-	-/-	0.39/1.56
*E. aerogenes* (ATCC 13045)	2000/-	1000/-	1000/-	1000/-	2000/-	0.19/0.19
*P. stuartii* (ATCC 29916)	-/-	/-	-/-	500/-	500/-	0.09/0.19
*P. mirabolis*	-/-	8000/-	500/-	-/-	-/-	25/50
*K. pneumoniae*	-/-	4000/-	-/-	-/-	-/-	25/50
*S. flexneri*	-/-	8000/-	500/-	-/-	-/-	25/50
*S. typhi* (ATCC 6539)	4000/-	8000/-	-/-	-/-	-/-	0.09/6.25
*S. paratyphi* A	-/-	8000/-	-/-	-/-	-/-	0.19/6.25
*S. paratyphi* B	-/-	4000/-	500/-	-/-	-/-	0.19/0.19
Gram (+)						
*E. faecalis* (ATCC 10541)	-/-	-/-	-/-	-/-	-/-	0.19/1.56
*S. aureus* (ATCC 25922)	-/-	4000/-	-/-	-/-	-/-	0.19/0.78

-, MIC or MBC was greater than 8000 μg/ml). *C E* Crude extract, *n-hex F* n-hexane fraction, *EA F* Ethyl acetate fraction, *n-but F* n-butanol fraction, *Res F* Residue fraction, *Rif* Rifampicin

#### Antibacterial properties of compounds isolated from P. fulva stem barks

Although the crude extract and fractions showed poor antibacterial activities, the isolated compounds generally demonstrated more or less interesting activities (Table 2). These activities varied with the tested species and within the same species of *E. coli,* it varies with strains. The most sensitive bacterium was *P. aeruginosa* (PA01) on which all the tested compounds were active with MICs ranging from 6.25 to 400 μg/ml while the less susceptible microorganisms were *E. coli* ATCC11775 and *S. flexneri* (Table 2). Moreover, the activities varied with the tested compound. Compounds **2**, **3**, **4**, **5**, **6** and **7** were the most active (MICs = 6.25 to 400 μg/ml) while compound **10** showed the least antibacterial activities (MICs = 100 to 400 μg/ml) (Table 2).

**Table 2:** MICs and MBCs (μg/ml) of isolated compounds on tested bacterial strains

Bacteria	1	2	3	4	5	6	7	8	9	10	11	Rif
Gram (-)												
*P. aeruginosa* (PA01)	25/50	6.25/6.25	6.25/12.5	6.25/12.5	6.25/12.5	6.25/-	6.25/-	50/50	100/-	400/-	400/-	0.19/1.56
*P. aeruginosa* (ATCC 27853)	100/-	100/-	50/-	50/-	-/-	50/-	50/-	-/-	50/-	400/-	400/-	25/50
*E. coli* (ATCC 8739)	-/-	12.5/12.5	25/50	12.5/-	-/-	-/-	25/50	25/50	-/-	-/-	25/50	0.09/6.25
*E. coli* (ATCC 10536)	-/-	100/-	100/-	100/-	100/-	25/50	100/-	100/-	100/-	100/-	-/-	0.19/0.78
*E. coli* (ATCC 11775)	-/-	-/-	-/-	-/-	-/-	100/-	-/-	-/-	50/100	-/-	-/-	0.39/1.56
*E. aerogenes* (ATCC 13045)	50/100	25 /-	50/-	25/400	-/-	-/-	100/-	12.5/400	25/400	200/-	-/-	0.19/0.19
*P. stuartii* (ATCC 29916)	-/-	200/-	-/-	-/-	12.5/-	100/-	100/200	100/400	25/100	100/100	400/-	0.09/0.19
*P. mirabolis*	100/-	100/-	-/-	-/-	-/-	100/200	100/-	200/-	50/-	400/-	400/-	25/50
*K. Pneumoniae*	100/-	12.5/200	12.5/-	6.25/400	6.25/-	100/-	100/-	25/-	100/-	100/-	100/-	25/50
*S. flexneri*	-/-	-/-	-/-	100/-	-/-	100/-	-/-	-/-	-/-	-/-	-/-	25/50
*S. typhi* (ATCC 6539)	25/100	25/200	25/-	25/-	50/-	-/-	100/200	100/-	50/100	-/-	400/400	0.09/6.25
*S. paratyphi* A	100/-	25/25	-/-	50/200	400/400	100/200	100/200	50/400	50/100	100/200	50/100	0.19/6.25
*S. paratyphi* B	-/-	12.5/25	25/-	25/-	25/-	100/200	100/100	100/200	25/50	200/-	-/-	0.19/0.19
Gram (+)												
*E. faecalis* (ATCC 10541)	25/50	6.25/12.5	6.25/12.5	6.25/12.5	6.25/12.5	50/200	50/200	12.5/25	25/50	-/-	-/-	0.19/1.56
*S. aureus* (ATCC 25922)	-/-	12.5/100	25/-	25/400	50/-	25/400	25/400	25/200	25/25	100/-	12.5/400	0.19/0.78

*Rif* Rifampicin, -: MIC or MBC was greater than 400 μg/ml

### Radical scavenging activity-50 (RSa50) of the crude extract, fractions and compounds from *P. fulva* stem barks

Generally, the tested samples from *P. fulva* scavenged more than 50% of the DPPH free radical in solution. The strongest antioxidant effect was observed with the crude extract, having RSa50 values of 84.86 μg/ml. The highest DPPH free radical scavenging activity of the isolated compounds was recorded from **7** with RSa50 = 135.39 μg/ml. However, their activities were less than that of the reference drug (RSa50 = 58.29 μg/ml) eventhough the difference was not significant (P˃0.05) (Table 3).

**Table 3:** Antioxidant activity of the crude extract, fractions and compounds from the stem bark of *Polyscias fulva* (IC 50 in μg/ml)

Test substance	DPPH test	β- Carotene/ linoleic acid
1	1141.10 ± 0.05^e^	14.77 ± 0.49^a^
3	nd	98.09 ± 9.32^b^
7	135.39 ± 3.33^abc^	108.13 ± 17.59^b^
8	180.08 ± 83.07^bc^	291.68 ± 77.58^c^
9	277.55 ± 77.28^d^	nd
10	190.82 ± 74.30^cd^	nd
11	1961.90 ± 0.05^f^	768.24 ± 0.03^f^
Hexane fraction	nd	660.73 ± 21.52^e^
Crude extract	84.86 ± 0.00^a^	128.52 ± 0.00^b^
Ethyl acetate fraction	93.65 ± 0.00^ab^	91.21 ± 0.00^b^
n-butanol fraction	100.58 ± 0.00^ab^	128.04 ± 0.00^b^
L-ascorbic acid	58.29 ± 0.25^a^	386.26 ± 1.09^d^

Along each column, values with the same letter superscripts are not significantly different. Waller Dunkan (*p* < 0.05). nd: not determined (RSa >2000 μg/ml)

Concerning the free radicals arising from the oxidation of the linoleic acid, the most active of all the tested samples was **1** with RSa50 = 14.77 μg/ml, followed by the ethyl acetate fraction (RSa50 = 91.21 μg/ml), **3** (RSa50 = 98.09 ± 9.32 μg/ml), **7** (RSa50 = 108.13 μg/ml), *n*-butanol fraction (RSa50 = 128.04 μg/ml), crude extract (RSa50 = 128.52 μg/ml) and **8** (RSa50 = 291.68 μg/ml). All these samples were more active than the reference drug (L-ascorbic acid) which had RSa50 value of 386.26 μg/ml (Table 3).

None of the tested samples presented superoxide anion scavenging activity.

## Discussion

### In vitro antibacterial activities

The crude dichloromethane-methanol (1:1 v/v) extract, fractions and isolated compounds from *P. fulva* stem bark displayed varied antibacterial activities on the studied pathogens. The fractionation process may concentrate active compounds in some fractions (ethyl acetate and *n*-butanol fractions) relatively more active as compared to the crude. In most active fractions, the fractionation could also have reduced antagonistic effects among compounds [15].

The relative wide range of antibacterial properties for the crude extract and fractions can be explained by the presence of various classes of potentially active secondary metabolites detected in them. Indeed saponins [16], phenols [17], tannins [18] and alkaloids [19], identified in thesetested materials [9] have been reported to possess antimicrobial activities. The relative wide range of antimicrobial properties may results from the individual or from the combined modes of action of compounds belonging to the identified groups of constituents. Alkaloids have the ability to fix in between the components of DNA molecules, inhibit DNA synthesis through topoisomerase inhibition [20] thus inhibiting microbial growth while tanins complex proteins via non-specific forces such as hydrogen, hydrophobic and covalent bindings [21]. The ability of tannins to complex enzymes and other membranous proteins of microorganisms inhibit their growth. The inhibition of microbial growth by phenolic compounds may be due to iron deprivation or hydrogen binding with vital proteins such as microbial enzymes [22]. These potentially active compounds may have gained the interior of microorganisms through specific receptors at their surfaces before exerting their antimicrobial activities as observed in this study. The various groups of identified contituents, with their combined modes of action, should have resulted to high antimicrobial activities of the tested extract and fractions. But the antimicrobial activities of these extract and fractions were moderate, suggesting the need of compound isolation in order to void likely antagonistic effects among constituents of the plant extract and fractions.

Compounds **1** and **3** are phenolic compounds. Then, the observed antibacterial activities could be attributed to the presence of hydroxyl groups [15]. Compound **2** (Steroid) presented antibacterial activities. This activity was also observed with compounds **4** (Triterpene) and **5** to **11**, triterpenoids saponins with the same basic skeleton. It is therefore not surprising since some individual triterpenes have shown this type of biological activities [23]. Compound **5** with the same basic skeleton as compound **4** presented less antibacterial activity. This could be due to the substitution of the hydroxyl group in compound **4** by a (3-*O*-[*α*-L-rhamnopyranosyl (1–2)-*α*-L-arabinopyranosyl] group in compound **5**. This later is less active on bacteria compared to compound **6**. In fact, compound **5** is lacking a hydroxyl group present in counpound **6** at position 16. This additional group may be responsible for that increase in the activity. The compound **7** is more active on all the tested microorganisms than compound **6**. This difference in activity may be attributed to the transfer of hydroxyl group from position 16 in the compound **6** to the position 23 in the compound **7** and also to the absence of the 3-*O*-[*α*-L-rhamnopyranosyl] group in this compound **7**. Compound **8** that result from the addition of the 3-*O*-[*α*-L-rhamnopyranosyl] group to compound **7** is less active on bacteria. This modification may have slightly reduced the antibacterial activity of the compound **8** eventhough both compounds have large spectrum compared to all the others which have medium activity on the tested microorganisms. The substitution of the (3-*O*-[*α*-L-rhamnopyranosyl (1–2)-*α*-L-arabinopyranosyl] present in compound **8** by a 3-*O*-(methyl-*β*-D-glucurono-pyranosiduronoate) group followed by the absence of hydroxyl group at the position 23 in the compound **9** may be responsible for the reduction of the antibacterial activity in this latter. Compound **10** in which the 3-*O*-(methyl-*β*-D-glucurono-pyranosiduronoate) group was replaced by the (3-*O*-[*α*-L-rhamnopyranosyl (1–2)-*α*-L-arabinopyranosyl] followed by the addition of a hydroxyl group at position 23 and of the [*O*-*α*-L-rhamnopyranosyl (1–4) -*O*-*β*-D-glucopyranosyl group at the position 6 of the glucopyranosyl group is less active against bacteria compared to compound **9**. This may be due to the observed modifications. As far as compound **11** is concerned, the *α*-L-4-*O*-acetyl group added at position 6 of the rhamnopyranosyl linked to the 28-*O* (1–4)-*β*-D-glucopyranosyl-(1–6)-*β*-D-glucopyranosyl] group may have slightly reduced its antibacterial activities.

The variation of activities with the tested species and strains could be ascribed to the difference in their genetic constitution. Analysis of the minimum inhibitory concentrations, minimum bactericidal concentrations obtained with respect to crude extract, fractions and isolated compounds on some microorganisms revealed that the ratios MBC/MIC were less or equal to 4. Values of these ratios less or equal to 4 indicate that the tested samples are bactericidal [24].

### Antioxidant activities of the crude extract, fractions and compounds

Their broad range of free radicals effects in biological systems has drawn the attention of many experimental works [25]. In the present study, the antioxidant properties of crude extract, fractions and compounds from the stem barks of *P. fulva* was demonstrated on DPPH and on free radical arising from linoleic acid oxidation. Phenolic compounds and saponins isolated from this plant may be partialy responsible for the radical scavenging activities of the crude extract and fractions [26]. The ethyl acetate and *n*-butanol fractions, the most active against free radicals (DPPH and that from oxidation of linoleic acid) contained compound **7** (saponin) and **1** (phenol). These two compounds were among the most effective on the tested free radicals and can then be considered as highly responsible for the observed activities. The antioxidative effects of phenolic and saponin components are mainly due to their redox properties, which can play an important role in absorbing and neutralizing free radicals, quenching singlet and triplet oxygen, or decomposing peroxides [27]. It is known that the antioxidant activity of a compound is proportional to the number of hydroxyl groups it contains [28]. This probably explains the high radical scavenging activities of compounds especially compounds **1** and **3** which are more efficient in protecting the linoleic acid from oxidation than the reference drug. Furthermore, lignans are good antioxidants scavenging free radicals that may play a role in some diseases [29].

## Conclusion

This work shows that *P. fulva* possesses antibacterial and antioxidant properties and constitute a step forward in the possible standardization of an antimicrobial phytomedicine with a wide range spectrum of activity from *Polyscias fulva* isolated compounds.
